# Frustrations of supported catalytic clusters under operando conditions predicted by a simple lattice model

**DOI:** 10.1038/s41598-022-21534-4

**Published:** 2022-10-11

**Authors:** Alexander Korobov

**Affiliations:** Materials Chemistry Department, V. N. Karazin Kharkiv National University, Kharkiv, 61022 Ukraine

**Keywords:** Heterogeneous catalysis, Reaction kinetics and dynamics, Statistical mechanics, Surface chemistry, Nanoparticles, Nonlinear phenomena

## Abstract

The energy landscape with a number of close minima separated by low barriers is a well-known issue in computational heterogeneous catalysis. In the framework of the emerging out-of-equilibrium material science, the navigation through such involved landscapes is associated with the functionality of materials. Current advancements in the cluster catalysis has brought and continues to bring essential nuances to the topic. One of them is the possibility of frustration of the catalytic centre under operando conditions. However, this conjecture is difficult to check either experimentally or theoretically. As a step in this direction, as-simple-as-possible lattice model is used to estimate how the supposed frustrations may couple with the elementary reaction and manifest themselves at the macroscopic scale.

## Introduction

Currently we are witnessing significant advancements in the field of heterogeneous catalysis with small clusters and single atoms^1–38^. Basic expectations include essentially new catalytic processes, enhanced selectivity of known important processes, atom saving, etc. High sensitivity of these catalysts to the environmental conditions is promising from the angle of their subtle tuning. Another side of the coin is the stability issue.

Also we are witnessing tremendous advancements in the field of out-of-equilibrium materials^39–48^. Basic expectations include life-like adaptive efficient intelligent materials and systems due to functionality distributed across all scales. Various applications require such materials with dynamic dissipative properties that evolve with time. These include systems powered by chemical fuels and orchestrated through chemical reaction networks. General principles of simulation and design of these materials are just emerging as results and experience accumulate in various related fields. The enzyme catalysis is surely one of these fields with its ambitious plans to develop complex chemically fuelled catalytic networks endowed with Darwinian properties^48^.

Intuitively, one more closely related field is the cluster catalysis, though considerable efforts are required to properly reveal and formalize numerous aspects of relevance. One important step is to treat a catalyst in terms of "structure—function" rather than just "structure—property". This creates, in particular, a broader context for considering the above "flexibility—stability" dilemma, which is generally inherent in out-of-equilibrium materials: the more adaptive the more unstable. To promote the longevity of a material means to find ways towards its self-repair and dynamical robustness^44^.

Generally, quite different behaviour is implied when using terms flexibility, fluxionality, reconstruction, reshaping, (dynamic) evolution, dynamic structural transformations, etc. This may be cluster isomerization^1^, disintegration^2^, sintering^2,10^, phase transition^13,15^. The common here is the departure from the idea of the catalytic site as a static atomic configuration, which has been dominating in the field since this concept of catalytic site was introduced^22^. It is rooted in the understanding that the structure and behaviour of a catalytic site may be fairly sensitive to the temperature, pressure, support, reactants, intermediates, and products. As a result, catalytic sites may undergo dynamic structural transformations under operando conditions in response to changes in the environment.

The above transformations extend over different length scales. The lowest one is the scale of single atoms and small clusters. Cluster catalysts are highly fluctuating, and their shapes can be affected by the size and surrounding conditions. The dynamic effects are expected to be the most pronounced for clusters consisting of a few tens of atoms^13,14,16,17^.

In the first-principle perspective this means that the energy landscape (consisting of a large number of close minima separated by low-energy barriers) and discrete electronic structure of a cluster, involved in themselves, depend in complex ways on the environment. These dependencies are fairly subtle and difficult to measure or simulate. In particular, frustrations caused by a degenerate energy landscape is an intriguing possibility that can be overlooked. From general considerations, most stable clusters are not obligatory most catalytically active, or even at all active. And indeed, metastable isomers with higher energies have recently been shown to be more active^14,18,19^. Though less populated, metastable states may play a sound role. Accordingly, corresponding regions of energy landscapes need to be taken into consideration.

How this structural dynamics may couple with elementary reactions? The time scale of structural dynamics is generally believed to be somewhat greater than that of elementary reactions^23–25^. This assumes a sequential implementation of events. First a catalytic cluster takes a suitable active configuration under particular operando conditions. Then an elementary reaction occurs, during which this cluster is static. Even in this case the catalyst dynamics may have nontrivial effects on catalysis.

Very recent literature provides examples when the time scale of the dynamic evolution of catalyst structures overlaps with that of chemical reactions. In^13^ this is linked with the solid-to-liquid phase transitions induced by adsorption; in^14,15^ liquid-to-solid phase transition occurs. This assumes a concurrent implementation of events. The catalytic centre is dynamic during the chemical reaction.

How common is this option? Two considerations seem to be relevant in answering this question.

First, the displacement of only few atoms of the catalytic cluster (say, two or three of ten) may be sufficient to provide the concerted realization of an elementary reaction. In this case the coincidence of time scales seems to be realistic.

Second, more significant, it is logically possible that an elementary reaction occurs as a result of the reshaping of a cluster. E.g. an adsorbed molecule initiates the reshaping (transition from one minimum of the energy landscape to another), and conditions favourable for the reaction appear during this reshaping. Note that in this case the reshaping rather than chemical reaction determines the common time scale. This means, in particular, that the adequate supply with reagents is more realistic.

Quite logically, parallels with enzyme catalysis appear more and more in the literature on cluster catalysis. In living organisms enzymes belong to the lowest scale at which an enzyme knows how to behave without instructions from upper levels. Being large and flexible, enzymes usually sample their conformational space slowly in comparison with reaction rates. Still the question about the coupling of the degrees of freedom remains disputable with various arguments pro et contra^12,13,16^. Two nuances are worth noting in this connection. First, a "hard copy" of the transition state may be mimicked for some enzymes. But they are ineffective without accompanying protein dynamics^12^. Secondly, only part of reactant particles supplied to an active centre of any nature are converted into product particles. In the case of enzyme catalysis the percentage of converted species is very high just due to the internal degrees of freedom. This gives grounds to consider cluster dynamics as an important factor of high catalytic efficiency.

In exploring the realm of fluctuating catalytic clusters, one of the main concerns is their stability. Intuitively, more labile species are expected to be more catalytically active as a result of higher sensitivity to environmental conditions. But doubts naturally arise about their ability to function stably under harsh reaction conditions, which is mandatory for a real catalyst. Understanding the evolutional behaviour of fluxional catalytic clusters is considered as an indispensable step in providing their stable functioning^2^.

Note that the term stability may generally refer quite different situations. In the simplest case there is one stable catalytic isomer at operando conditions. The next, more involved, situation is the set of isomers provided that the Boltzmann distribution is established at operando conditions and maintained during the reaction. The next complication step assumes the availability of metastable isomers which may be less populated but much more active. Ultimately this line leads to catalytically active dissipative systems. In this context the term "stability" has two different meanings: the stability of the structure of a supported catalyst and the stability of the behaviour of a catalytic system at operando conditions. To distinguish these two meanings, the term "robust" will be used in the latter case. Intuitively, more dynamic catalytic system is less robust. But when studying such complex systems, intuition can fail.

With this in mind, the issue of robustness was previously addressed in terms of very simple lattice model of the reversible reshaping of supported metal nanoparticles under reaction conditions^49,50^. In that model reaction induced catalytic sites appear and disappear depending on the operando conditions. The main conclusion is that the model demonstrates the possibility to keep the robust dynamic behaviour of the system in spite of the high lability of its structure. It should be noted, however, that the catalytic sites of that model had no internal degrees of freedom.

The present paper deduces an as-simple-as-possible lattice model endowed with internal degrees of freedom to simulate frustrations due to which the supported catalytic cluster is dynamic during the chemical reaction. Main points to be approached with this model are as follows. In what way, if at all, internal degrees of freedom may manifest themselves at the mesoscopic scale. Whether any collective effects are generally possible in the system. Whether the robust functioning of the system is possible and, if so, under which conditions.

## Model

The Langmuir—Hinshelwood mechanism is implied for the reaction A + B → P, which means that both particles A and B need catalytic activation to react. Within the conventional framework the catalyst activates A and B independently of each other. To simulate the situation when the time scale of the dynamic evolution of catalyst structures overlaps with that of chemical reactions (the catalytic centre is dynamic during the chemical reaction) the model is endowed with two pairs of close minima separated with low-energy barriers.

Geometric and electronic structures of the initial cluster C make it indifferent to B whereas A can be uptake and activated, though not too easily and quickly (e.g. this may be 2D → 3D transition). Newly formed cluster CA can uptake but not sufficiently activate B. The peculiarity of the model is two practically equal minima for CAB in the energy landscape which are separated by a relatively low barrier. This sets the stage for frustrations. The transition from one metastable state to another creates favourable conditions for reaction to occurs. One more peculiarity is the capability of cluster CA to easily uptake second particle A with the formation of CA_2_ which is more active with respect to B. Cluster CA_2_B also has two close minima separated with even lower barrier (one more stage for frustrations). Accordingly, reaction in this case is faster.

**Figure 1:**
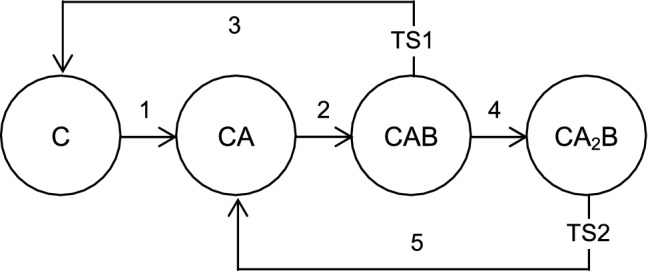
Graph representation of the suggested model; C denotes cluster, A and B denote reagent particles; TS denotes transition state.

This functioning of the catalytic centre can be represented as a graph (Fig. 1). In spite of apparent simplicity it includes four routs resulting in product formation, *r*_1_: 1-2-3, *r*_2_: 1-2-4-5, *r*_3_: 2-3, and *r*_4_: 2-4-5. It can operate in kinetic, diffusion and mixed regimes, and in the mixed regimes it converts constant supply of reagents into oscillating release of products. Such behaviour is known to be a consequence of the complexity of the chemical reaction, which is based on the underlying non-linearity of the equations used to describe the kinetics^51^. Accordingly, it is completely impossible in the case of simple static catalytic centre.

Generally, the graph as a model for the catalytic centre provides the "structure—function" rather than just "structure—property" description. Note that the reaction stoichiometry is A:B = 1:1. The second species A is required to keep the catalytic centre in its most active state. In this sense it may be considered as a fuel. Generally, such energy-reach metastable states are considered as an efficient tool of quick adaptation of out-of-equilibrium systems. When A is deficient, the catalytic centre is switched to a less efficient regime.

**Figure 2:**
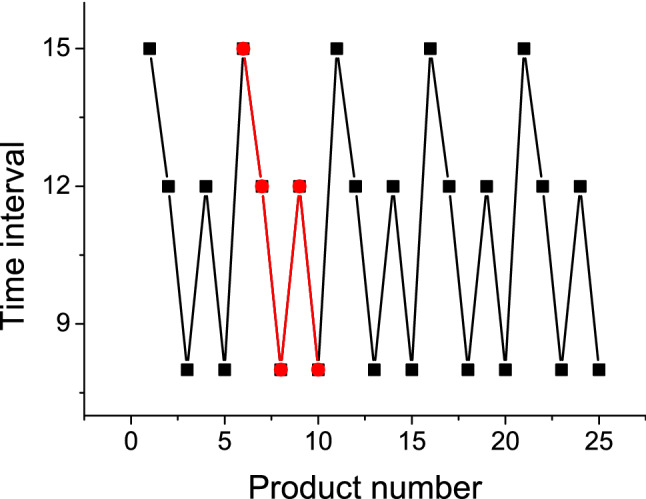
If supply is stationary with τ_*s*_ = 11 beats, intervals τ_P_ between two successive releases of product particles will be [-15-12-8-12-8-]…; repeated fragment is shown in red.

The oscillating regimes are better illustrated with an arbitrary numerical example. The cluster is assumed to have an internal clock (an internal metronome). Let the duration of longer route *r*_1_ equals τ_1_ = 15 beats and that of the shortest route τ_4_ = 5. Then τ_*s*_ ≥ 15 corresponds to diffusion region, τ_*s*_ ≤ 5 corresponds to kinetic region, and 5 ≤ τ_*s *_*≤ *15 corresponds to mixed region, where τ_*s*_ is the supply time of particles A. We are interested in the period τ_P_ between two successive releases of product particles P. In the diffusion region it will be equal to τ_*s*_; the cluster will fire every 15 beats and then wait for the next particle A for another (τ_*s*_—15) beats (this is indicated with w in Table 1). In the kinetic region τ_P_ = 5 irrespectively of τ_*s*_ provided no poisoning occurs (this aspect is discussed below). In the mixed region there are nine (τ_1_-τ_4_ + 1) different oscillation regimes each of which includes two other routes, *r*_2_ and *r*_3,_ with durations τ_2_ = 12 and τ_3_ = 8 respectively (Fig. 2 shows one regime for τ_*s*_ = 11 as an example; all regimes are listed in Table 1). Only three routes participate in each regime, either *r*_1_, *r*_2_, *r*_3_ or *r*_2_, *r*_3_, *r*_4_. Note that in all cases the average value of τ_P_ equals to τ_*s*_.

**Table 1:** All possible regimes of the cluster operation in the case of arbitrary selected numerical example: τ_s_ is the supply time, τ_P_ is the period between two successive releases of P, $\overline{\tau }_{P}$ is the averaged value, τ_1_ = 15, τ_2_ = 12, τ_3_ = 8 , τ_4_ = 5 (beats), *r*_i_ are routs on the graph.

			τ_s_	τ_P_	$\overline{\tau }_{P}$
Regime	Diffusion	r_1_, r_2_, r_3_	17	[15-w-w]-15-w-w….	17
			16	[15-w]-15-w…	16
			15	15–15-15–15-15–15…	15
	Mixed		14	[15–15-15–15-15–12-8-w-w-w…]	14
			13	[15–15-12–8-w-w-…]	13
			12	[15–12-8-w…]	12
			11	[15–12-8–12-8…]	11
			10	15-[12–8-12–8-12–8…]	10
		r_2_, r_3_, r_4_	9	12-[8–12-8–12-5]…	9
			8	12-[8–12-5–8-12–5-5–8-12–5]…	8
			7	12–5-[8–12-5–5-5]…	7
			6	12–5-[5–5-8–12]…	6
	Kinetic		5	12-[5–5-5–5-5-…	
			4	12-[5–5-5–5-5-…	
			3	12-[5–5-5–5-5-…	
			2	12-[5–5-5–5-5-…	
			1	12-[5–5-5–5-5-…

Main regularities of cluster functioning may be summarized as follows.

- *r*_1_ is the longest rout in which only one species A is used; start and finish at C
- *r*_2_ is a shorter route which assumes the use of two species A; start at C, finish at CA
- *r*_3_ even more shorter route which is realized when the second species A is late; start at CA*,* finish at C
- *r*_4_ is the shortest route which is realized when the second species A is timely; start and finish at CA
- *r*_3_ may follow *r*_2_ or *r*_3_ (if the second A is late) and finish at C; this may be followed by *r*_2_ or *r*_1_;
- *r*_4_ may follow only *r*_2_ and, in turn, may be followed by either *r*_4_ or *r*_3_.

Different choice of particular time intervals will change details but general features remain (such as transformation of constant supply into oscillating regimes and sensitivity of regimes to the supply).

We are interested in the possibility of revealing the internal degrees of freedom from the observed kinetic behaviour. The very fact that the constant supply is transformed into various oscillating regimes of product formation indicates their existence. Formally, the complete set of oscillation regimes provides information about their quantitative characteristics. But exact supply of isolated cluster can hardly be done in practice. Hence, the inverse problem is of purely formal interest. Therefore, the next question is the behaviour of array of identical clusters under real inflow regimes. In particular, the possibility of collective behaviour is of particular interest.

## Results and discussion

**Figure 3:**
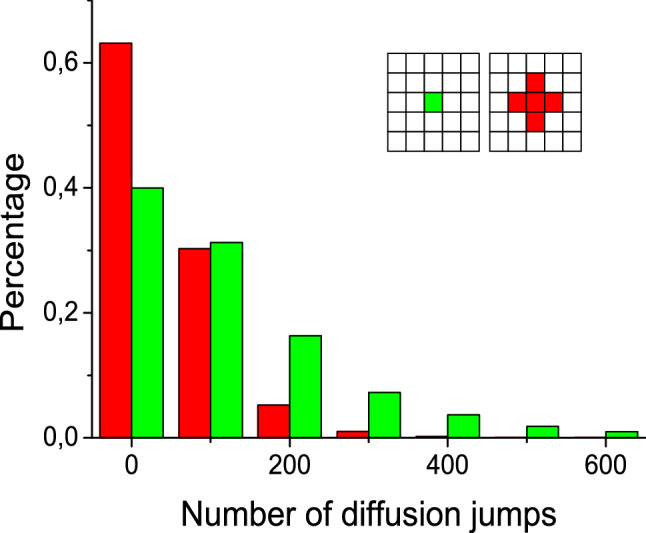
The histogram compares the numbers of diffusion jumps on 11 × 11 lattice that randomly adsorbed particle needs to reach the cluster in the centre; the cluster size is the only difference, one (green) or five (red) cites; averaged over 10^6^ runs.

Though surface diffusion is known to be the source of numerous surprises, in kinetic Monte Carlo simulations it is frequently considered as the fastest process and does not get too much attention. In the present context, a more detailed elaboration is needed. As a starting point, Fig. 3 shows results for the simplest possible model: one adsorbed particle within the 11 × 11 capture zone with the cluster in its centre. The cluster may be represented by one or five lattice cites as shown in the insert. One particle A is adsorbed at random within the capture zone. Then one of four neighbour cites is selected at random and the particle is moved their. This continues until the particle reaches the cluster. The number of diffusion jumps is counted. Figure 3 shows results for 10^6^ runs.

In the computational heterogeneous catalysis the routine is to represent the active centre by one cite on the lattice. In Fig. 3 this case is shown with green. The distribution of the number of jumps has fairly long tail. If an active centre (cluster) possess the border of definite length, the uptake of adsorbed particles is more efficient and, accordingly, the distribution is much more narrow. The average numbers of jumps are 131 and 51 respectively. With this in mind we will further assume that cluster has the border of a certain length.

In passing to numerous particles A and A + B (1:1) in the capture zone, the next question is about the mutual influence of reactant particles A and B. Within the above model the cluster starts when first particle A arrived. This is simulated as follows. The *L*x*L* capture zone is randomly filled with adsorbate particles, either only A or A and B (1:1); the cluster itself and its four-cite border are free from adsorbate. When the pre-assigned coverage θ_s_ is reached, the diffusion starts and runs until first particle A is registered at the border. The number of diffusion jumps is counted for each adsorbate particle. Time is counted in Monte Carlo steps (MCS). One MCS is time during which each lattice cite is visited once on the average; 1MCS = *L*x*L*. Table 2 shows mean values and ranges of MCS at which first A particle reaches the border, the percentages of arrivals at first MCS, the corresponding values for the number of diffusion jumps, and percentages of various options for filling the border with adsorbate particles (termed here border configurations). The table contains two coverages, θ_s_ is the substrate coverage and θ_c_ is the coverage corresponding to the particular configuration.

**Table 2:** Results of the kinetic Monte Carlo simulations of the surface diffusion on 11 × 11 lattice; started with certain coverage of the substrate and free border, finished when A is registered at the border; averaged over 10^6^ runs; θ_s_ is the substrate coverage, θ_c_ is the coverage corresponding to particular configuration, configuration means specific filling of the border with adsorbate (only configurations with A are included), MCS is the Monte Carlo step at which run stops, 1-st step (%) is the percentage of runs finished at first MCS, Jumps is the number of diffusion jumps made by particle A before reaching the border.

θ_s_:		0.2		0.45		0.9	
		A only	A + B	A only	A + B	A only	A + B
Config	θ_c_	%					
000A	0.25	82.4	65.7	59.2	38.8	27.0	14.1
00AA	0.50	16.2	5.6	32.8	9.8	43.9	11.3
00AB		.0	24.0	.0	33.3	.0	27.9
0AAA	0.75	1.3	.2	7.5	1.0	25.0	3.2
0AAB		.0	1.2	.0	4.7	.0	11.5
0ABB		.0	3.1	.0	10.4	.0	19.8
AAAA	1.00	.0	.0	.6	.0	4.0	.3
AAAB		.0	.0	.0	.2	.0	1.2
AABB		.0	.1	.0	.6	.0	2.9
ABBB		.0	.1	.0	1.2	.0	7.9
**MCS**							
Mean		2.5	5.8	1.4	2.9	1.05	2.2
Range		1 ÷ 39	1 ÷ 96	1 ÷ 11	1 ÷ 63	1 ÷ 5	1 ÷ 174
1-st step (%)		46.5	26	75.6	47	95.2	69.5
**Jumps**							
Mean		3.3	7.2	1.5	2.7	1.03	1.2
Range		1 ÷ 46	1 ÷ 121	1 ÷ 16	1 ÷ 59	1 ÷ 5	1 ÷ 24

Table 2 shows results averaged over 10^6^ runs. In some cases it is convenient to treat these sequential runs as simultaneous functioning of 10^6^ clusters. Thus, it may be said that in the case of coverage θ_s_ = 0.9 with particles A as many as 95% of clusters start at first MCS, nearly all clusters start within three MCS, and absolutely all clusters start within five MCS. The number of jumps equals the number of MCS; i.e. only one diffusion jump occurs at each MCS on the average. The border configuration 00AA dominates followed by 000A and 0AAA almost equally.

When the coverage is decreased, the number of starts at first MCS also decreased. In the case of θ_s_ = 0.2 the same 95% of clusters start within eight MCS with dominating border configuration 000A. The range of MCS is also eight times bigger.

**Figure 4:**
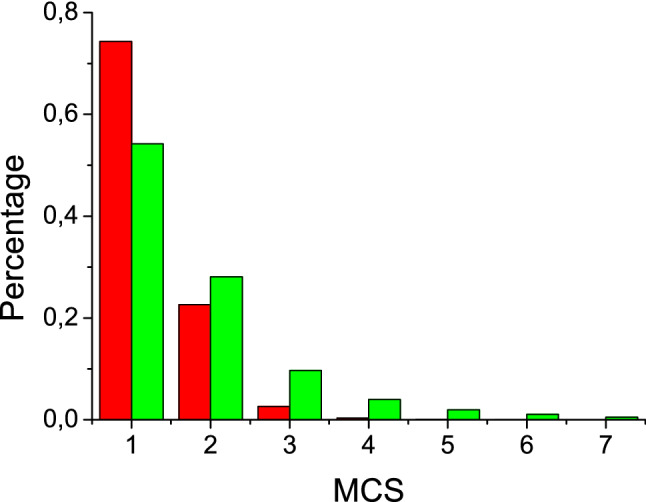
The histogram compares the numbers of Monte Carlo steps on 11 × 11 lattice that particle A needs to reach the four-cite border of cluster in the case of 0.45 substrate coverage with A only (red) and 0.9 substrate coverage with A + B (green); averaged over 10^6^ runs.

It is informative to compare two cases, θ_s_ = 0.45 with A only and θ_s_ = 0.9 with A + B (Table 2 , Fig. 4). In both cases the surface concentration of A is the same, but in the latter case particles B are also present, reducing considerably the number of free cites for diffusion jumps. (Note that lateral interactions are not considered here). As a result, the mean MCS is increased, the range of MCS is the biggest in the table, the mean number of diffusion jumps is considerably smaller than that of MCS (i.e. the jump of A does not occur at every step because of the lack of free space).

We also need to understand how the coverage of the cluster border evolve with time. The simulations start, as before, from the capture zone with preassigned coverage and adsorbate free four-cite border. The configurations of the border are registered during 10^6^ MCS. Results are given in Table 3. Unlike the previous table, all 15 configurations are included in the consideration. At small coverages of the capture zone the adsorbate-free border strongly dominates, followed by configurations with θ_c_ = 0.25. On the contrary, at high coverages the percentage of configurations with θ_c_ = 1 exceeds 65%. For a given coverage of the capture zone the sum of percentages of all configurations multiplied by corresponding θ_c_ gives the averaged coverage of the border θ_b_. As seen from the table, θ_b_ and θ_s_ well coincide in all cases.

**Table 3:** Results of the kinetic Monte Carlo simulations of the surface diffusion on 11 × 11 lattice; started with certain coverage of the substrate with A + B (1:1) and free four-cite border, continued during 10^6^ runs; θ_s_ is the substrate coverage, θ_c_ is the coverage corresponding to particular configuration, configuration means specific filling of the border with adsorbate (all possible configurations are included), θ_b_ is the averaged coverage of the border, "Σ without A" is the total percentage of configurations without A, Changeability is the percentage of MCS at which the configuration is changed.

Border config	θ_s_:	0.1	0.5	0.9
	θ_c_	%		
OOOO	0.00	62.3	5.6	.0
OOOA	0.25	15.6	11.9	.2
OOOB		15.9	11.6	.2
OOAA	0.50	1.4	9.3	1.2
OOAB		2.9	19.0	2.5
OOBB		1.4	9.4	1.1
OAAA	0.75	.1	3.2	3.8
OAAB		.2	9.8	10.9
OABB		.2	10.0	11.1
OBBB		.1	3.2	3.4
AAAA	1.00	.0	.4	4.0
AAAB		.0	1.7	16.8
AABB		.0	2.7	24.5
ABBB		.0	1.6	16.6
BBBB		.0	.5	3.8
Σ without A		74.7	30.3	8.7
Σ without B		74.9	30.4	9.1
θ_b_		0.11	0.51	0.90
Changeability (%)		41	75	34

One more characteristic needed is how frequently the configuration of the border is changed (changeability or life time of configurations). In Table 3 it is given as the percentage of MCS at which the configuration is changed. Any change counts; e.g. one particle A leaves the border and another comes to a different position. The coverage θ_s_ = θ_b_ = 0.5 provides the highest dynamics. At smaller coverages the decrease of dynamics is because of the lack of adsorbed particles; at bigger coverages the decrease of dynamics is because of the lack of free cites for diffusion jumps.

Note also that the total percentage of configurations without A (or B) is rather big at θ_s_ = 0.5 and is fairly big at θ_s_ = 0.9. This leads to the question of how such border dynamics is combined with the internal dynamics of the cluster.

To answer this question, the above simulation algorithm is completed by the ability of the cluster to uptake reagents from its border. As before, the cluster is represented by the central cite of the 11 × 11 lattice and its border consists of four neighbour cites. First of all, this means the need to relate the time determined by diffusion and the internal time of the cluster. To begin with, one beat of the cluster is equated to one MCS. Remind that the cluster uptakes reagents only at definite beats. In the stationary regime particles A are consumed at each fifth step, i.e. 2, 7, 12, 17, … Then particles B are consumed at steps 5, 10, 15, …; also at each fifth step. Thus, B is consumed three steps after A and next A is consumed two steps after B. Substrate coverage is kept constant. The expectation was that this stationary functioning may be ensured under certain conditions. Surprisingly, it was not justified: the cluster works at best several hundreds of MCS; the mean MCS value is only 20.4. Details are given in Table 4, column 2. The cluster stops because of the lack of A or B particles at the border at the required time; both causes occur equally often (which indicates, among other things, the quality of computations). The uptake of reagents by the cluster doubled the changeability of the border but considerably decreased its coverage in comparison with substrate coverage. The result is fairly big percentage of configurations without A (or without B), which causes a quick stop of the cluster. Two obvious possibilities to improve this situation is to forbid (or restrict) the outflow of adsorbed particles from the border and to enlarge the border.

**Table 4:** Results of the kinetic Monte Carlo simulations; previous algorithm is completed by the ability of cluster to uptake reagents from its border; particles A are consumed at each fifth step, particles B are also consumed at each fifth, three steps after A; substrate coverage is kept constant; designations as in Table 3.

	No uptake	Uptake of A and B by cluster			
	Four-cite border			Eight-cite border	
		Outflow +	Outflow –	Outflow +	Outflow –
	1	2	3	4	5
**Stop due to**					
–Lack of A (%)		44	46	51	51
–Lack of B (%)		56	54	49	49
**MCS stop**					
– Mean	–	20.4	36.1	188.2	191.8
– Range	–	2 ÷ 225	2 ÷ 402	2 ÷ 1452	2 ÷ 1462
Changeability (%)	34	77	50	70	41
θ_b_	0.90	0.73	0.89	0.85	0.95
Σ without A (%)	8.4	16.3	10.7	–	–
Σ without B (%)	9.3	16.0	10.9	–	–

Till now the border does not differ from the rest of substrate. It is logical to test a different case when the cluster keeps the adsorbed particles at the border. Once hitting the border, they cannot diffuse back to the substrate. Corresponding results are given in column 3 of Table 4. Again we see opposite tendencies, border coverage is increased up to that of substrate but the changeability is decreased. The resulting gain is insignificant and left no chance for the stationary functioning of the cluster. It is important to emphasize that among all configurations without A as many as 4.6% make configurations BBBB; for configurations AAAA the percent is the same. This means that in about 10% of all cases the cluster irreversibly stops because of the poisoning of the border with one of reagents.

Formally, one more possibility to provide the stationary functioning of the cluster is to enlarge its border. It should be noted that eight-cite border is somewhat too big for small clusters which usually have several ten atoms all in all. Still, this possibility has been tested with both allowed and forbidden outflow from the border to the substrate. Results are summarized in columns 4 and 5 of Table 4. Note that MCS values are practically the same in both cases in spite of fairly different border coverage and changeability. And though they are about twenty times greater than values for four-cite border, they are two small to talk about stationary functioning of the cluster.

The above considerations lead to two conclusions.

Fluctuations are inherent in small systems under study, which makes the stationary supply of the cluster with reagents very difficult, if not impossible. Rather, stop-and-restart regimes control the efficiency of the catalytic system as a whole.

Though the systems without outflow of reagents from the border are more efficient at initial stages, the irreversible poisoning of the border by one of reagents is the inevitable outcome.

Simulation details of the stop-and-restart regime are as follows. Each run counting 10^6^ MCS starts, as before, from the adsorbate free border (8 or 4 cites) and the capture zone with preassigned coverage, which is kept constant throughout the computation. The cluster in the centre of the capture zone starts working when first particle A appears at the border. Once the lack of required A (or B) is detected at a certain MCS, the cluster is switched to standby mode until this particle appear at the border. The result is two sequences, the sequence of working intervals and the sequence of pauses. Short intervals dominate in both cases, and this is more pronounced for pauses. An obvious characteristic of the efficiency of the catalytic system is the total duration of the working periods *w*_t_ expressed as the percentage of the run duration.

**Figure 5:**
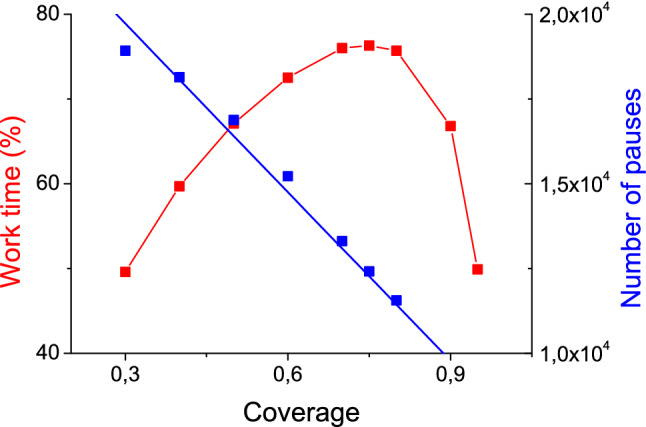
Coverage-dependent properties of the catalytic system under study; results of kinetic Monte Carlo simulations on 11 × 11 lattice with four-cite border averaged over 10^6^ runs.

There are two results common to all computations. The lack of A and the lack of B at the border at required time equally often cause the cluster to stop. If the outflow of reagents from the border to the substrate is forbidden, the ultimate outcome is the irreversible poisoning of the catalyst. The rest of results is somewhat different for eight-cite and four-cite borders. The efficiency of former systems are nearly insensitive to the substrate coverage: *w*_t_ = 92% in the case of θ_s_ = 0.9 and *w*_t_ = 91% in the case of θ_s_ = 0.5; the numbers of pauses are about 10^4^ and 4⋅10^4^ respectively. On the contrary, catalytic systems with four-cite borders demonstrate expressed sensitivity to the substrate coverage as illustrated by Fig. 5 and Table 5. The number of pauses is linearly decreased with the increase of coverage (Fig. 5, right axis), but the averaged duration of pauses pass through the minimum. Correspondingly, the efficiency of the system passes through the maximum (Fig. 5, left axis). Note that on the left of the maximum free border cites dominate whereas on the right the border is occupied with two and more adsorbed particles (either A or B). In the first case the lack of required particle is associated with the insufficient coverage of the border (which is always less than substrate coverage); in the second case the lack of required particle is associated with the excess of particles of another kind. Maximum is determined by the balance of these two types of configurations. This reflects the fact that the lack of vacant cites for diffusion is as material as the lack of diffusing particles.

**Table 5:** Results of kinetic Monte Carlo simulations on 11 × 11 lattice with four-cite border averaged over 10^6^ runs; θ_s_ is the substrate coverage, θ_b_ is the averaged coverage of the border, *c* is the percentage of border configurations with two or more A or B, *w*_t_ is the total duration of the working periods expressed as percentage of the run duration.

θ_s_	θ_b_	*c*(%)	*w*_t_(%)
0.3	0.25	8.9	49.6
0.4	0.30	16	59.7
0.5	0.40	25.8	67.1
0.6	0.48	38.8	72.5
0.7	0.55	54.1	76.0
0.8	0.65	73.9	75.7
0.9	0.80	91.1	66.8
0.95	0.88	97.8	49.9

It follows that for the system under study the optimal substrate coverage is determined by the interplay between the supply of adsorbed particles to the border and their mobility within the border. And under no circumstances should the 76% efficiency be surpassed.

One more question is to what extent this result depends on the internal clock of the cluster. Through the previous part of the paper the 5-5-2 time intervals were used: particle A is consumed at each fifth step, particle B is also consumed at each fifth step and two steps after particle A. Computations show that time interval between particles does not affect the efficiency of the system. The efficiency is somewhat bigger for the slower clock: the triple increase of Δτ from 5 to 15 MCS results in the increase of efficiency from 76 to 92%. This gain is difficult to qualify as significant.

## Summary

The presence of several close minima separated with relatively low barriers in the energy landscape significantly complicates the computational study of heterogeneous catalysts, especially if free energy landscapes are concerned. Nevertheless, the interest in catalytic systems with such landscapes has increased significantly in recent years. This, in particular, is because the navigation through complex landscapes is associated with the functionality of a system in the framework of the emerging out-of-equilibrium material science. Partial transfer of the functionality to the material level is a tempting though very challenging perspective.

Recent progress in understanding the cluster catalysis add some essential nuances to the topic. One of them is the possibility of frustration of the catalytic centre under operando conditions. However, this conjecture is difficult to check either experimentally or theoretically. In addition to conventional computational difficulties, it needs to be taken into account that clusters covered with reactants may differ in shape and properties from clusters covered with products, which considerably complicates the energy landscape.

While the dynamic nature (fluxionality) of clusters has confidently taken its place at the atomic scale of the overall picture, at the mesoscopic scale the routine is still to represent the catalytic centre as a single static cite (denoted in models as * or z). Consequently, even existing atomic-level ideas about the internal degrees of freedom of the catalytic centre are not translated to the next level.

Above considerations justify the following approach. Assume that the supported catalytic cluster has internal degrees of freedom. Also assume that they provide the background for frustrations under operando conditions. The question is whether the internal degrees of freedom will manifest themselves at the mesoscopic scale. Whether it is possible to determine (discern, guess) them in the kinetic experiment. What mathematical tools are needed for data processing?

The non-stationary (oscillating) outflow of products in response to the stationary inflow of reagents is an indicator of complexity of a catalytic system. However, using this indicator, it is necessary to clarify what exactly is the "stationary inflow". When small supported clusters are concerned, the supply chain includes three stages, each with a pitfall. The starting point is the stationary inflow of stoichiometric gaseous reagent mixture to the catalyst surface. Reagents have to adsorb at the surface. The success or failure of the adsorption act is known to depend on features of the approach of the molecule to the surface (steering effect)^52^. The same surface may be more hospitable to molecules of one kind and less hospitable to molecules of another kind. The orientation of the molecule relative to the surface also matters. Hence, a stationary inflow of the gaseous phase of reagents does not yet guarantee the stationary inflow to the capture zone. Once the stationary coverage of the capture zone with the reagent mixture is provided in one or another way, the next stage is the diffusion towards the border of the cluster. The diffusion coefficient may differ considerably from one experimental or computational estimation to another^12^. Even bigger problem is that at high coverages, that are of interest in catalysis, diffusion coefficients depend on coverages. Hence, equal diffusion coefficients for both reagents is a zero approximation. The final stage is a subtle balance at the border of the cluster between inflow, outflow and uptake of reagent molecules.

The above considerations concern the Langmuir—Hinshelwood mechanism. If we turn to Eley–Rideal mechanism, one more important factor is added. According to this mechanism one reactant molecule, say A, is preadsorbed within the capture zone of the catalytic cite, as described above. The second molecule, say B, approaches the catalytic cite directly from the gaseous phase. Sufficient supply of A requires the capture zone of a certain size. This determines, in turn, the density of catalytic cites at the surface. On the other hand, sufficient supply of B requires a certain (rather high) density of catalytic cites which restricts the size of capture zones. Thus, the size of the capture zone becomes a compromise in the supply of A and B.

Within this general framework, one of conclusions in this work is that the stationary supply of small supported clusters with reagent molecules is unrealistic because of fluctuations inherent in small systems, even if above problems with adsorption and diffusion within the capture zone are avoided. Accordingly, there are no grounds to expect any collective behaviour. Instead, the stop and restart operation is the inherent regime. Its efficiency is characterized by the portion of time when the cluster is working. It is largely determined by the consistency of the two time scales, internal cluster time and supply time. In particular, the ease of restart after each pause is desirable.

When internal degrees of freedom couple with the elementary reaction in such a way that the cluster is dynamic during reaction, this is manifested at the mesoscopic scale as the dependence of the efficiency of catalytic system on the coverage passing through a maximum. In the suggested model frustrations provide this agreement of time scales. Note that this case is outside the scope of established computational methods.

The model that suggests the above features of the catalytic system under study is a lattice model in which the frustrated cluster is represented as a graph. It is rooted in generic experimental and theoretical findings and is simplified as far as possible to still represent internal degrees of freedom and proper coupling with the elementary reaction. No contradictions incompatible with common sense and available knowledge have been registered in exploring the model. All this gives grounds to believe that the results presented are of a fairly general character.

Of course, the system complexity is not an end in itself. Enzymatic catalysis provides an example of the great efficiency of each catalytic act due to conformational dynamics. Current information on the vide spectrum of behaviour of supported clusters under operando conditions gives grounds to expect, in some cases, a similar use of the cluster conformational dynamics. This, in turn, gives rise to the problem of revealing and controlling the internal degrees of freedom. Hopefully, the results presented are useful in this respect and will prompt further theoretical and experimental steps.

## Data Availability

The datasets used and/or analysed during the current study available from the corresponding author on reasonable request.
